# Synthesis and crystallographic characterization of 6-hydroxy-1,2-dihydropyridin-2-one

**DOI:** 10.1107/S205698902300974X

**Published:** 2023-11-14

**Authors:** Sara K. Phillips, Savannah G. Brancato, Samantha N. MacMillan, Mark J. Snider, Andrew J. Roering, Katherine A. Hicks

**Affiliations:** a Department of Chemistry, The State University of New York at Cortland, Cortland, New York 13045, USA; b Department of Chemistry and Chemical Biology, Cornell University, Ithaca, New York 14853, USA; c Department of Chemistry, The College of Wooster, Wooster, Ohio 44691, USA; Texas A & M University, USA

**Keywords:** crystal structure, hydrogen bonding, nicotinic acid derivative

## Abstract

The title compound, C_5_H_5_NO_2_, is a hy­droxy­lated pyridine ring that has been studied for its involvement in microbial degradation of nicotinic acid. Here we describe its synthesis as a formic acid salt, rather than the standard hydro­chloride salt that is commercially available, and its spectroscopic and crystallographic characterization.

## Abstract 2

The synthesis of the title compound as a formic acid salt, rather than the standard hydro­chloride salt that is commercially available, and its spectroscopic and crystallographic characterization are described.

## 1. Chemical context

6-Hydroxy-1,2-dihydropyridin-2-one, more commonly known as 2,6-dihydroxypyridine (2,6-DHP), is a derivative of nicotinic acid, a common compound found within personal care products (Behrman & Stanier, 1957 ▸; Hicks *et al.*, 2016 ▸; Nakamoto *et al.*, 2019 ▸). Recent work has focused on the bacterial hydrolysis of nicotinic acid for use in bioremediation efforts (Bokor *et al.*, 2022 ▸). Synthesis of 2,6-DHP can be accomplished by reaction between 2,6-di­chloro­pyridine and potassium *tert*-butoxide to afford 2,6-di-*tert*-but­oxy­pyridine (**1**) followed by reaction with formic acid to produce the product **2** as the pyridone tautomer (Scheme 1; Kocienski, 1994 ▸). The identification of **2** was confirmed by ^1^H, ^13^C and IR spectroscopy. The ^1^H NMR spectrum suggested a non-symmetric pyridone mol­ecule with an N—H proton at δ = 11.47 ppm. The aromatic region of the spectrum suggested that each of the three protons on the aromatic backbone of **2** were in different chemical environments highlighted by their different chemical shifts of δ = 7.66, 6.91 and 6.60 ppm. These shifts, along with their splitting patterns and coupling constants, are consistent with the structure of **2**. IR spectroscopic data of **2** were also consistent with the overall structure of a pyridone tautomer. Crystals of **2** were grown from slow evaporation of a saturated methanol solution. The solid-state structure of **2** was consistent with the solution state as the title mol­ecule crystallized as the keto tautomer. 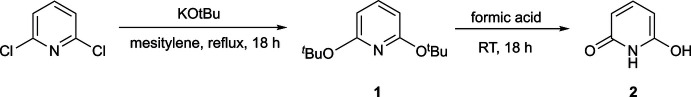


## 2. Structural commentary

**Figure 1:**
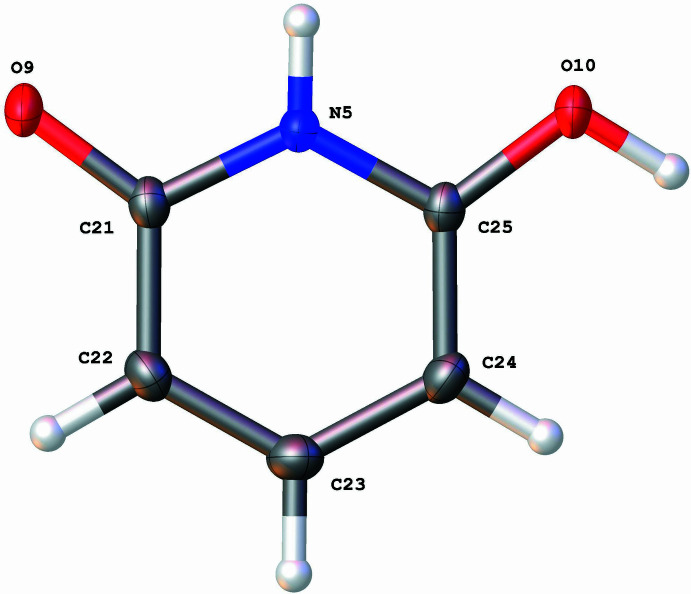
A view of **2** showing the atom-numbering scheme for one independent mol­ecule. Displacement ellipsoids are drawn at the 50% probability level.

The structure of 2,6-di­hydroxy­pyridine (Fig. 1 ▸) shows the expected 2,6-disubstitution of the pyridine ring. The bond lengths and angles are routine for nitro­gen-containing aromatic compounds (Table 1 ▸).

## 3. Supra­molecular features

**Figure 2:**
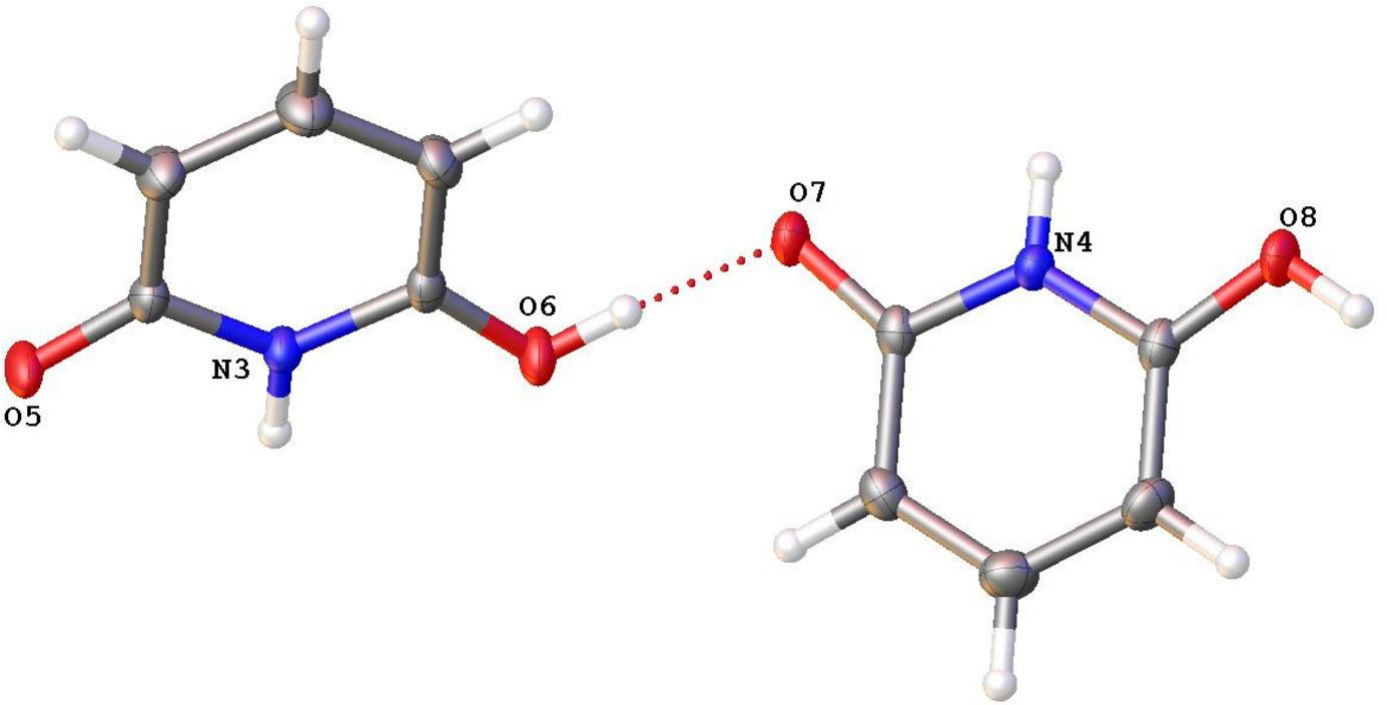
A view of the inter­molecular inter­actions in **2**.

**Figure 3:**
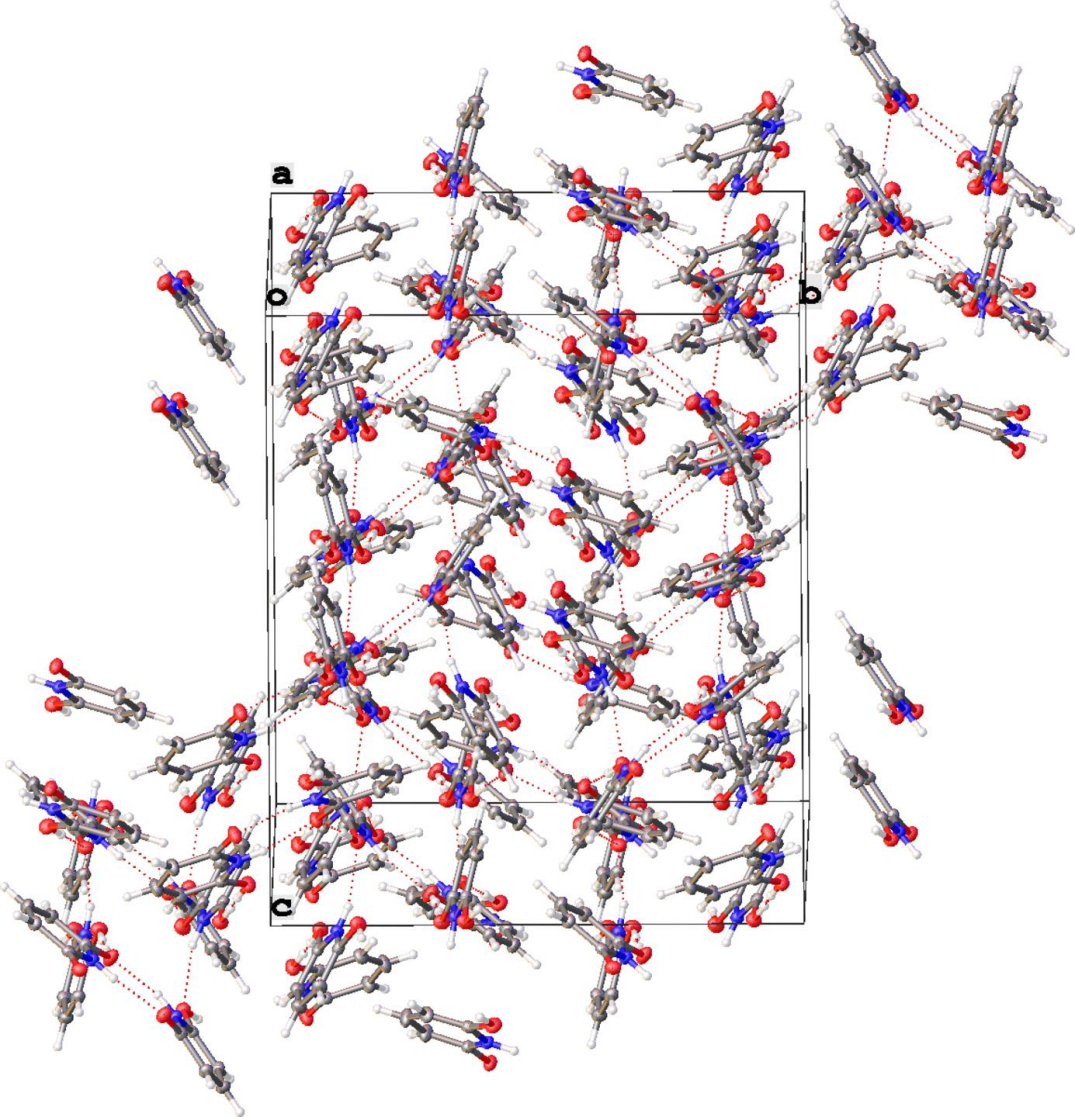
A view of the mol­ecular packing in **2**.

There are six independent mol­ecules in the asymmetric unit of **2**; of these, two pairs of mol­ecules are each held together by O—H⋯O hydrogen bonds. In both instances, the H atoms in the hydrogen bonds are disordered over two positions with refined occupancies of 0.51 (3) and 0.49 (3) at the O6 and O7 sites, respectively, and 0.39 (3) and 0.61 (3) at the O2 and O3 sites, respectively. The mol­ecules pack together in the solid state with inter­molecular O—H⋯O and N—H⋯O inter­actions (Table 2 ▸ and Fig. 2 ▸). The crystal packing of the title compound involves no π–π ring inter­actions (Fig. 3 ▸).

## 4. Hirshfeld surface analysis

**Figure 4:**
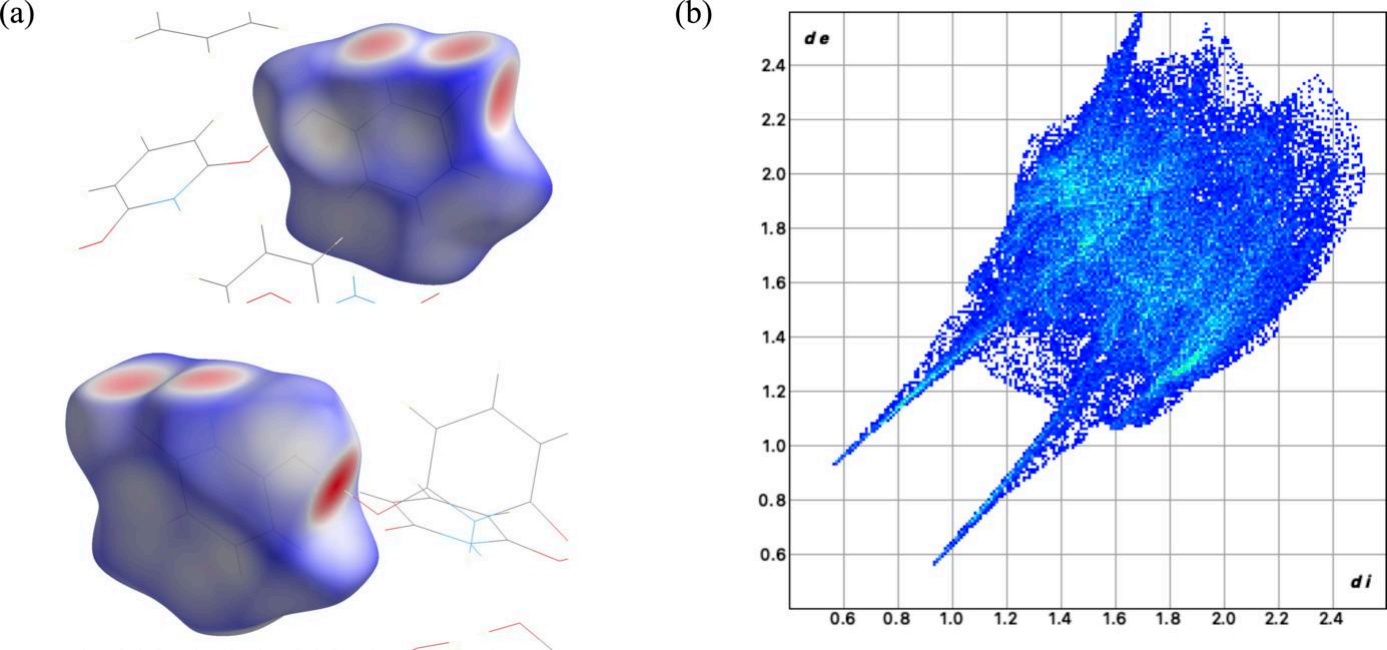
(*a*) Hirshfeld surface representations of **2** with the function *d*_norm_ plotted onto the surface indicating the *E*—H⋯O (*E* = N, O) inter­actions; (*b*) two-dimensional fingerprint plot.

The Hirshfeld surface analysis of **2** was performed and the associated two-dimensional fingerprint plots were generated using *Crystal Explorer 21.5* software (Spackman *et al.*, 2021 ▸). Visualizations used a red–white–blue color scheme where red indicates shorter contacts, while blue indicates longer contacts. There are four red spots on the *d*_norm_ surface (Fig. 4 ▸ *a*) and these spots indicate the direction and strength of the inter­molecular *E*—H⋯O (*E* = N, O). The two-dimensional fingerprint plots are shown in Fig. 4 ▸ *b*. The resulting fingerprint plot indicates strong O⋯H inter­actions, as shown by the two prominent spikes on either side of the diagonal. The N⋯H inter­actions are shown in the ‘wings’ of the plot and are not as prominent as the O⋯H inter­actions.

## 5. Database survey

A search for the pyridone tautomers of relatively simple dihy­droxy-substituted pyridines in the Cambridge Structure Database (CSD version 5.44, last update April 2023; Groom *et al.*, 2016 ▸) revealed 23 crystal structures. Nearly all these structures have N—H⋯O and O—H⋯O hydrogen-bonding motifs, similar to those observed in the title compound. The structures with dissimilar motifs involve inter­molecular inter­actions with solvent mol­ecules or intra­molecular hydrogen bonding. The closest analogues to **2** were found to be GUBKIZ and NOQGOR (Gerhardt & Bolte, 2015 ▸); these structures contain N—H⋯O and O—H⋯O(solvent) hydrogen-bonding motifs.

## 6. Synthesis and crystallization

**1**: A 100 mL round-bottom flask equipped with a stir bar was charged with 2,6-di­chloro­pyridine (1.00 g, 6.80 mmol, 1 eq) and 15 mL of mesitylene solvent. To the solution was added potassium *tert*-butoxide (1.52 g, 13.6 mmol, 2.1 eq). The solution was then refluxed under N_2_ for 18 h. A color change from colorless to deep red was observed. After 18 h, the solution was allowed to cool to room temperature and the solution was washed with water (3 × 20 mL). The organic layer was collected, dried over sodium sulfate and used without purification in step 2.

**2**: To the crude solution from step 1 in a 20 mL scintillation vial was added formic acid (1.00 mL, 17.8 mmol, 2.6 eq). The bi-layered solution was stirred in air at high speed for 18 h when a solid precipitate formed. The solid was collected and dried under vacuum to yield 0.180 g (17% over 2 steps).

^1^H NMR (300 MHz, ppm), 11.47 (*bs*, 1H NH), 7.68 (*t*, 1H), 6.91 (*d*, 1H), 6.60 (*d*, 1H). ^13^C NMR (75 MHz, ppm), 163.7, 147.0, 142.2, 114.9, 108.5. IR (cm^−1^): 1596 *m*, 1333 *m*, 825 *w*, 772 *w*, 706 *s*.

Crystals suitable for X-ray analysis were grown from slow evaporation of a saturated methanol solution. The melting point of 2,6-DHP was measured at 460–465 K.

## 7. Refinement

Crystal data, data collection and structure refinement details are summarized in Table 3 ▸. All non-hydrogen atoms were refined anisotropically. Hydrogen atoms bonded to carbon were included in calculated positions and refined using a riding model. Hydrogen atoms bound to N and O were located in the difference-Fourier map, and refined semi-freely with the help of distance restraints.

## Supplementary Material

Crystal structure: contains datablock(s) I. DOI: 10.1107/S205698902300974X/jy2036sup1.cif

Structure factors: contains datablock(s) I. DOI: 10.1107/S205698902300974X/jy2036Isup2.hkl

The SI contains NMR and IR spectroscopy data of the titled compound. DOI: 10.1107/S205698902300974X/jy2036sup3.docx

Supporting information file. DOI: 10.1107/S205698902300974X/jy2036sup4.mol

Supporting information file. DOI: 10.1107/S205698902300974X/jy2036Isup5.cml

CCDC reference: 2306392

Additional supporting information:  crystallographic information; 3D view; checkCIF report

## Figures and Tables

**Table 1:** Selected geometric parameters (Å, °) for **2**

O9—C21	1.2789 (12)	N5—C21	1.3688 (12)
O10—C25	1.3193 (12)	N5—C25	1.3557 (12)
			
C25—N5—C21	124.86 (9)	N5—C21—C22	116.60 (8)
C21—N5—H5	117.3 (8)	O10—C25—N5	114.23 (8)
C25—N5—H5	117.8 (8)	O10—C25—C24	126.58 (9)
O9—C21—N5	117.12 (9)	N5—C25—C24	119.19 (9)
O9—C21—C22	126.28 (9)		

**Table 2:** Hydrogen-bond geometry (Å, °) for **2**

*D*—H⋯*A*	*D*—H	H⋯*A*	*D*⋯*A*	*D*—H⋯*A*
O2—H2*A*⋯O3	0.84 (2)	1.60 (2)	2.4317 (10)	174 (4)
N1—H1⋯O9^i^	0.88 (1)	1.98 (1)	2.8606 (11)	175 (1)
O3—H3⋯O2	0.87 (2)	1.57 (2)	2.4317 (10)	176 (3)
O4—H4⋯O5^ii^	0.981 (14)	1.500 (14)	2.4768 (10)	173.5 (14)
N2—H2⋯O1^iii^	0.89 (1)	1.89 (1)	2.7554 (11)	166 (1)
O6—H6⋯O7	0.86 (1)	1.58 (1)	2.4381 (10)	177 (3)
N3—H3*B*⋯O11^iv^	0.87 (1)	1.95 (1)	2.8155 (11)	170 (1)
O7—H7*A*⋯O6	0.88 (2)	1.56 (2)	2.4381 (10)	179 (3)
O8—H8*A*⋯O9^v^	0.91 (1)	1.58 (1)	2.4803 (10)	175 (1)
N4—H4*A*⋯O10^i^	0.87 (1)	2.07 (1)	2.8999 (11)	161 (1)
O10—H10⋯O11^iv^	0.95 (1)	1.52 (1)	2.4690 (9)	176 (1)
N5—H5⋯O2^i^	0.88 (1)	1.90 (1)	2.7699 (11)	167 (1)
O12—H12*A*⋯O1^iii^	0.90 (1)	1.61 (1)	2.5057 (10)	178 (2)
N6—H6*A*⋯O5^vi^	0.88 (1)	1.91 (1)	2.7893 (11)	172 (1)

Symmetry codes: (i) 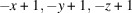 ; (ii) 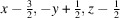 ; (iii) 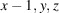 ; (iv) 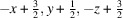 ; (v) 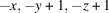 ; (vi) 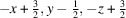 .

**Table 3:** Experimental details

Crystal data	
Chemical formula	C_30_H_30_N_6_O_12_
*M*_r_	666.60
Crystal system, space group	Monoclinic, *P*2_1_/*n*
Temperature (K)	100
*a*, *b*, *c* (Å)	9.58785 (4), 16.83642 (8), 19.55978 (10)
β (°)	103.7319 (5)
*V* (Å^3^)	3067.19 (3)
*Z*	4
Radiation type	Cu *K*α
μ (mm^−1^)	0.97
Crystal size (mm)	0.38 × 0.12 × 0.10

Data collection	
Diffractometer	XtaLAB Synergy, Dualflex, HyPix
Absorption correction	Gaussian (*CrysAlis PRO*; Rigaku OD, 2021 ▸)
*T*_min_, *T*_max_	0.453, 1.000
No. of measured, independent and observed [*I* > 2σ(*I*)] reflections	83138, 6554, 6155
*R*_int_	0.035
(sin θ/λ)_max_ (Å^−1^)	0.635

Refinement	
*R*[*F* ^2^ > 2σ(*F* ^2^)], *wR*(*F* ^2^), *S*	0.032, 0.083, 1.02
No. of reflections	6554
No. of parameters	477
No. of restraints	14
H-atom treatment	H atoms treated by a mixture of independent and constrained refinement
Δρ_max_, Δρ_min_ (e Å^−3^)	0.19, −0.35

Computer programs: *CrysAlis PRO* (Rigaku OD, 2021 ▸), *SHELXT2014/5* (Sheldrick, 2015*a* ▸), *SHELXL2016/6* (Sheldrick, 2015*b* ▸), and *OLEX2* (Dolomanov *et al.*, 2009 ▸).

## supplementary crystallographic information

### Crystal data

**Table:**

C_30_H_30_N_6_O_12_	*F*(000) = 1392
*M**_r_* = 666.60	*D*_x_ = 1.444 Mg m^−^^3^
Monoclinic, *P*2_1_/*n*	Cu *K*α radiation, λ = 1.54184 Å
*a* = 9.58785 (4) Å	Cell parameters from 59431 reflections
*b* = 16.83642 (8) Å	θ = 3.5–78.0°
*c* = 19.55978 (10) Å	µ = 0.97 mm^−^^1^
β = 103.7319 (5)°	*T* = 100 K
*V* = 3067.19 (3) Å^3^	Block, clear colourless
*Z* = 4	0.38 × 0.12 × 0.10 mm

### Data collection

**Table:**

XtaLAB Synergy, Dualflex, HyPix diffractometer	6554 independent reflections
Radiation source: micro-focus sealed X-ray tube, PhotonJet (Cu) X-ray Source	6155 reflections with *I* > 2σ(*I*)
Mirror monochromator	*R*_int_ = 0.035
Detector resolution: 10.0000 pixels mm^-1^	θ_max_ = 78.1°, θ_min_ = 3.5°
ω scans	*h* = −11→12
Absorption correction: gaussian (CrysAlisPro; Rigaku OD, 2021)	*k* = −21→21
*T*_min_ = 0.453, *T*_max_ = 1.000	*l* = −24→24
83138 measured reflections	

### Refinement

**Table:**

Refinement on *F*^2^	Primary atom site location: dual
Least-squares matrix: full	Hydrogen site location: mixed
*R*[*F*^2^ > 2σ(*F*^2^)] = 0.032	H atoms treated by a mixture of independent and constrained refinement
*wR*(*F*^2^) = 0.083	*w* = 1/[σ^2^(*F*_o_^2^) + (0.0411*P*)^2^ + 1.0736*P*] where *P* = (*F*_o_^2^ + 2*F*_c_^2^)/3
*S* = 1.02	(Δ/σ)_max_ = 0.001
6554 reflections	Δρ_max_ = 0.19 e Å^−^^3^
477 parameters	Δρ_min_ = −0.35 e Å^−^^3^
14 restraints	

### Special details

**Table:**

Geometry. All esds (except the esd in the dihedral angle between two l.s. planes) are estimated using the full covariance matrix. The cell esds are taken into account individually in the estimation of esds in distances, angles and torsion angles; correlations between esds in cell parameters are only used when they are defined by crystal symmetry. An approximate (isotropic) treatment of cell esds is used for estimating esds involving l.s. planes.

### Fractional atomic coordinates and isotropic or equivalent isotropic displacement parameters (Å^2^)

**Table:**

	*x*	*y*	*z*	*U*_iso_*/*U*_eq_	Occ. (<1)
O1	0.99326 (7)	0.14764 (4)	0.52810 (4)	0.02046 (15)	
O2	0.52056 (7)	0.20046 (5)	0.45281 (4)	0.02307 (16)	
H2A	0.433 (2)	0.190 (2)	0.449 (2)	0.028*	0.39 (3)
N1	0.75397 (8)	0.16938 (5)	0.49622 (4)	0.01657 (16)	
H1	0.7731 (13)	0.2085 (7)	0.4701 (6)	0.020*	
C1	0.86759 (10)	0.12574 (6)	0.53339 (5)	0.01703 (19)	
C2	0.83551 (11)	0.06266 (6)	0.57353 (5)	0.0215 (2)	
H2B	0.910124	0.031089	0.601141	0.026*	
C3	0.69311 (11)	0.04690 (6)	0.57242 (6)	0.0228 (2)	
H3A	0.671749	0.004190	0.599998	0.027*	
C4	0.58049 (10)	0.09106 (6)	0.53259 (5)	0.0196 (2)	
H4B	0.483773	0.078550	0.532377	0.024*	
C5	0.61270 (10)	0.15408 (6)	0.49300 (5)	0.01704 (19)	
O3	0.26881 (7)	0.17595 (5)	0.44883 (4)	0.02250 (16)	
H3	0.3589 (19)	0.1824 (14)	0.4497 (13)	0.027*	0.61 (3)
O4	−0.19312 (7)	0.12596 (5)	0.35615 (4)	0.02344 (16)	
H4	−0.2704 (15)	0.1017 (8)	0.3202 (7)	0.028*	
N2	0.04226 (8)	0.14771 (5)	0.39488 (4)	0.01710 (16)	
H2	0.0221 (13)	0.1564 (8)	0.4363 (6)	0.021*	
C6	0.18169 (10)	0.15386 (6)	0.39087 (5)	0.01790 (19)	
C7	0.21482 (11)	0.13432 (6)	0.32713 (6)	0.0213 (2)	
H7	0.310669	0.137636	0.321914	0.026*	
C8	0.10467 (11)	0.11005 (7)	0.27185 (6)	0.0239 (2)	
H8	0.126899	0.095290	0.228771	0.029*	
C9	−0.03761 (11)	0.10639 (6)	0.27684 (5)	0.0219 (2)	
H9	−0.111893	0.090907	0.237695	0.026*	
C10	−0.06756 (10)	0.12600 (6)	0.34062 (5)	0.01782 (19)	
O5	1.10984 (8)	0.44209 (4)	0.77157 (4)	0.02427 (17)	
O6	0.69473 (7)	0.46139 (4)	0.60703 (4)	0.02291 (16)	
H6	0.633 (3)	0.4403 (16)	0.5726 (11)	0.027*	0.51 (3)
N3	0.90320 (8)	0.44362 (5)	0.68705 (4)	0.01738 (17)	
H3B	0.8924 (13)	0.4921 (7)	0.7009 (7)	0.021*	
C11	1.02185 (10)	0.40333 (6)	0.72324 (5)	0.01834 (19)	
C12	1.03646 (12)	0.32439 (6)	0.70413 (6)	0.0254 (2)	
H12	1.116169	0.293498	0.727941	0.030*	
C13	0.93248 (13)	0.29207 (7)	0.64981 (6)	0.0295 (2)	
H13	0.942521	0.238376	0.636833	0.035*	
C14	0.81402 (11)	0.33486 (6)	0.61347 (6)	0.0240 (2)	
H14	0.744993	0.311212	0.576036	0.029*	
C15	0.79897 (10)	0.41320 (6)	0.63325 (5)	0.01727 (19)	
O7	0.51409 (7)	0.40623 (4)	0.50930 (4)	0.02113 (15)	
H7A	0.579 (3)	0.4265 (17)	0.5446 (12)	0.025*	0.49 (3)
O8	0.06505 (7)	0.32983 (5)	0.41128 (4)	0.02330 (16)	
H8A	−0.0264 (13)	0.3226 (8)	0.4150 (7)	0.028*	
N4	0.28349 (8)	0.37226 (5)	0.46665 (4)	0.01696 (17)	
H4A	0.3118 (13)	0.3515 (8)	0.4316 (6)	0.020*	
C16	0.38461 (10)	0.41017 (6)	0.51679 (5)	0.01732 (19)	
C17	0.33735 (11)	0.44811 (6)	0.57103 (5)	0.0218 (2)	
H17	0.402967	0.476182	0.606926	0.026*	
C18	0.19353 (12)	0.44388 (7)	0.57127 (6)	0.0260 (2)	
H18	0.161676	0.469269	0.608196	0.031*	
C19	0.09332 (11)	0.40380 (7)	0.51941 (6)	0.0239 (2)	
H19	−0.004815	0.401025	0.521012	0.029*	
C20	0.14144 (10)	0.36826 (6)	0.46563 (5)	0.01835 (19)	
O9	0.18597 (7)	0.69692 (4)	0.58239 (4)	0.02126 (15)	
O10	0.66116 (7)	0.66705 (4)	0.66883 (4)	0.01849 (15)	
H10	0.7367 (13)	0.6395 (8)	0.6998 (7)	0.022*	
N5	0.42153 (8)	0.67871 (5)	0.63253 (4)	0.01655 (16)	
H5	0.4396 (13)	0.7114 (7)	0.6004 (6)	0.020*	
C21	0.28056 (10)	0.66605 (6)	0.63264 (5)	0.01715 (19)	
C22	0.25389 (10)	0.61994 (6)	0.68815 (5)	0.0203 (2)	
H22	0.158220	0.610105	0.691582	0.024*	
C23	0.36839 (11)	0.58916 (6)	0.73752 (6)	0.0224 (2)	
H23	0.349750	0.558253	0.775009	0.027*	
C24	0.51056 (11)	0.60179 (6)	0.73441 (5)	0.0203 (2)	
H24	0.587906	0.579291	0.768459	0.024*	
C25	0.53506 (10)	0.64807 (6)	0.68018 (5)	0.01634 (18)	
O11	0.64103 (7)	0.09175 (4)	0.75468 (4)	0.01983 (15)	
O12	0.20548 (7)	0.07976 (5)	0.60556 (4)	0.02324 (16)	
H12A	0.1306 (14)	0.1044 (8)	0.5774 (7)	0.028*	
N6	0.42400 (8)	0.09381 (5)	0.67740 (4)	0.01662 (16)	
H6A	0.4165 (13)	0.0440 (7)	0.6899 (7)	0.020*	
C26	0.54837 (10)	0.13190 (6)	0.70929 (5)	0.01743 (19)	
C27	0.56380 (12)	0.21052 (6)	0.68974 (6)	0.0260 (2)	
H27	0.649710	0.238977	0.708997	0.031*	
C28	0.45219 (13)	0.24630 (7)	0.64195 (6)	0.0304 (3)	
H28	0.462424	0.300102	0.629325	0.036*	
C29	0.32527 (12)	0.20661 (7)	0.61155 (6)	0.0249 (2)	
H29	0.249173	0.232717	0.579442	0.030*	
C30	0.31366 (10)	0.12804 (6)	0.62962 (5)	0.01851 (19)	

### Atomic displacement parameters (Å^2^)

**Table:**

	*U*^11^	*U*^22^	*U*^33^	*U*^12^	*U*^13^	*U*^23^
O1	0.0114 (3)	0.0298 (4)	0.0185 (3)	0.0016 (3)	0.0002 (2)	0.0008 (3)
O2	0.0104 (3)	0.0268 (4)	0.0301 (4)	0.0005 (3)	0.0013 (3)	0.0117 (3)
N1	0.0118 (4)	0.0194 (4)	0.0174 (4)	−0.0001 (3)	0.0013 (3)	0.0032 (3)
C1	0.0133 (4)	0.0214 (5)	0.0148 (4)	0.0020 (3)	0.0000 (3)	−0.0032 (3)
C2	0.0195 (5)	0.0217 (5)	0.0210 (5)	0.0054 (4)	0.0002 (4)	0.0031 (4)
C3	0.0237 (5)	0.0199 (5)	0.0244 (5)	0.0007 (4)	0.0050 (4)	0.0050 (4)
C4	0.0157 (4)	0.0202 (5)	0.0229 (5)	−0.0015 (3)	0.0041 (4)	0.0014 (4)
C5	0.0127 (4)	0.0191 (4)	0.0181 (4)	0.0002 (3)	0.0013 (3)	−0.0003 (4)
O3	0.0113 (3)	0.0326 (4)	0.0218 (4)	−0.0029 (3)	0.0005 (3)	−0.0013 (3)
O4	0.0125 (3)	0.0331 (4)	0.0237 (4)	−0.0050 (3)	0.0022 (3)	−0.0064 (3)
N2	0.0123 (4)	0.0207 (4)	0.0177 (4)	−0.0014 (3)	0.0024 (3)	−0.0020 (3)
C6	0.0127 (4)	0.0177 (4)	0.0222 (5)	0.0001 (3)	0.0019 (3)	0.0020 (4)
C7	0.0164 (4)	0.0239 (5)	0.0247 (5)	0.0015 (4)	0.0069 (4)	0.0037 (4)
C8	0.0255 (5)	0.0271 (5)	0.0205 (5)	0.0003 (4)	0.0082 (4)	0.0002 (4)
C9	0.0205 (5)	0.0246 (5)	0.0187 (5)	−0.0031 (4)	0.0008 (4)	−0.0023 (4)
C10	0.0144 (4)	0.0167 (4)	0.0209 (5)	−0.0019 (3)	0.0011 (4)	−0.0001 (4)
O5	0.0181 (3)	0.0227 (4)	0.0261 (4)	0.0047 (3)	−0.0066 (3)	−0.0051 (3)
O6	0.0158 (3)	0.0241 (4)	0.0241 (4)	0.0013 (3)	−0.0048 (3)	−0.0018 (3)
N3	0.0143 (4)	0.0166 (4)	0.0193 (4)	0.0007 (3)	0.0001 (3)	−0.0023 (3)
C11	0.0146 (4)	0.0211 (5)	0.0179 (4)	0.0013 (3)	0.0010 (3)	0.0003 (4)
C12	0.0238 (5)	0.0218 (5)	0.0274 (5)	0.0069 (4)	−0.0004 (4)	−0.0019 (4)
C13	0.0319 (6)	0.0213 (5)	0.0319 (6)	0.0038 (4)	0.0007 (5)	−0.0073 (4)
C14	0.0225 (5)	0.0238 (5)	0.0227 (5)	−0.0027 (4)	−0.0002 (4)	−0.0059 (4)
C15	0.0133 (4)	0.0219 (5)	0.0158 (4)	−0.0022 (3)	0.0019 (3)	0.0006 (4)
O7	0.0119 (3)	0.0277 (4)	0.0218 (4)	−0.0025 (3)	0.0001 (3)	−0.0025 (3)
O8	0.0117 (3)	0.0300 (4)	0.0265 (4)	−0.0043 (3)	0.0012 (3)	−0.0042 (3)
N4	0.0128 (4)	0.0200 (4)	0.0176 (4)	−0.0006 (3)	0.0026 (3)	−0.0009 (3)
C16	0.0146 (4)	0.0174 (4)	0.0181 (4)	−0.0012 (3)	0.0001 (3)	0.0039 (3)
C17	0.0226 (5)	0.0223 (5)	0.0189 (5)	−0.0026 (4)	0.0020 (4)	−0.0014 (4)
C18	0.0267 (5)	0.0274 (5)	0.0261 (5)	−0.0009 (4)	0.0108 (4)	−0.0036 (4)
C19	0.0168 (4)	0.0271 (5)	0.0299 (5)	−0.0009 (4)	0.0093 (4)	−0.0003 (4)
C20	0.0134 (4)	0.0181 (4)	0.0224 (5)	−0.0010 (3)	0.0020 (4)	0.0037 (4)
O9	0.0110 (3)	0.0253 (4)	0.0252 (4)	−0.0008 (3)	−0.0002 (3)	0.0068 (3)
O10	0.0106 (3)	0.0242 (3)	0.0193 (3)	0.0011 (3)	0.0008 (2)	0.0022 (3)
N5	0.0120 (4)	0.0197 (4)	0.0171 (4)	0.0001 (3)	0.0017 (3)	0.0038 (3)
C21	0.0126 (4)	0.0175 (4)	0.0204 (5)	−0.0004 (3)	0.0020 (3)	−0.0008 (4)
C22	0.0152 (4)	0.0232 (5)	0.0237 (5)	−0.0004 (4)	0.0070 (4)	0.0016 (4)
C23	0.0236 (5)	0.0242 (5)	0.0207 (5)	0.0012 (4)	0.0077 (4)	0.0050 (4)
C24	0.0182 (5)	0.0231 (5)	0.0180 (5)	0.0041 (4)	0.0014 (4)	0.0036 (4)
C25	0.0132 (4)	0.0179 (4)	0.0168 (4)	0.0007 (3)	0.0013 (3)	−0.0022 (3)
O11	0.0143 (3)	0.0197 (3)	0.0214 (3)	−0.0014 (3)	−0.0040 (3)	0.0039 (3)
O12	0.0131 (3)	0.0304 (4)	0.0222 (4)	−0.0006 (3)	−0.0038 (3)	0.0036 (3)
N6	0.0130 (4)	0.0185 (4)	0.0166 (4)	0.0002 (3)	0.0000 (3)	0.0022 (3)
C26	0.0146 (4)	0.0203 (5)	0.0159 (4)	0.0001 (3)	0.0008 (3)	−0.0004 (4)
C27	0.0254 (5)	0.0202 (5)	0.0264 (5)	−0.0045 (4)	−0.0055 (4)	0.0018 (4)
C28	0.0369 (6)	0.0184 (5)	0.0291 (6)	−0.0001 (4)	−0.0058 (5)	0.0038 (4)
C29	0.0249 (5)	0.0234 (5)	0.0213 (5)	0.0067 (4)	−0.0045 (4)	0.0021 (4)
C30	0.0138 (4)	0.0254 (5)	0.0151 (4)	0.0031 (4)	0.0010 (3)	−0.0012 (4)

### Geometric parameters (Å, º)

**Table:**

O1—C1	1.2875 (12)	O7—H7A	0.879 (18)
O2—H2A	0.839 (19)	O7—C16	1.2858 (12)
O2—C5	1.2956 (12)	O8—H8A	0.905 (12)
N1—H1	0.880 (12)	O8—C20	1.3098 (12)
N1—C1	1.3708 (12)	N4—H4A	0.868 (12)
N1—C5	1.3655 (12)	N4—C16	1.3630 (12)
C1—C2	1.3978 (15)	N4—C20	1.3590 (12)
C2—H2B	0.9500	C16—C17	1.4026 (15)
C2—C3	1.3859 (15)	C17—H17	0.9500
C3—H3A	0.9500	C17—C18	1.3819 (15)
C3—C4	1.3879 (14)	C18—H18	0.9500
C4—H4B	0.9500	C18—C19	1.3946 (15)
C4—C5	1.3908 (14)	C19—H19	0.9500
O3—H3	0.867 (17)	C19—C20	1.3811 (15)
O3—C6	1.2928 (12)	O9—C21	1.2789 (12)
O4—H4	0.981 (14)	O10—H10	0.948 (11)
O4—C10	1.3096 (12)	O10—C25	1.3193 (12)
N2—H2	0.888 (12)	N5—H5	0.882 (12)
N2—C6	1.3616 (12)	N5—C21	1.3688 (12)
N2—C10	1.3556 (12)	N5—C25	1.3557 (12)
C6—C7	1.3971 (15)	C21—C22	1.4067 (14)
C7—H7	0.9500	C22—H22	0.9500
C7—C8	1.3818 (15)	C22—C23	1.3795 (14)
C8—H8	0.9500	C23—H23	0.9500
C8—C9	1.3919 (15)	C23—C24	1.3952 (14)
C9—H9	0.9500	C24—H24	0.9500
C9—C10	1.3850 (15)	C24—C25	1.3805 (14)
O5—C11	1.2856 (12)	O11—C26	1.2884 (12)
O6—H6	0.861 (14)	O12—H12A	0.897 (12)
O6—C15	1.2949 (12)	O12—C30	1.3139 (12)
N3—H3B	0.874 (12)	N6—H6A	0.881 (12)
N3—C11	1.3686 (12)	N6—C26	1.3671 (12)
N3—C15	1.3672 (12)	N6—C30	1.3622 (12)
C11—C12	1.3966 (14)	C26—C27	1.3955 (14)
C12—H12	0.9500	C27—H27	0.9500
C12—C13	1.3843 (15)	C27—C28	1.3810 (15)
C13—H13	0.9500	C28—H28	0.9500
C13—C14	1.3896 (15)	C28—C29	1.3919 (16)
C14—H14	0.9500	C29—H29	0.9500
C14—C15	1.3916 (15)	C29—C30	1.3805 (15)
			
C5—O2—H2A	117 (3)	C16—O7—H7A	114.1 (19)
C1—N1—H1	117.4 (8)	C20—O8—H8A	111.5 (9)
C5—N1—H1	117.1 (8)	C16—N4—H4A	117.0 (8)
C5—N1—C1	125.31 (9)	C20—N4—H4A	118.0 (8)
O1—C1—N1	116.42 (9)	C20—N4—C16	124.96 (9)
O1—C1—C2	126.71 (9)	O7—C16—N4	115.79 (9)
N1—C1—C2	116.88 (9)	O7—C16—C17	127.13 (9)
C1—C2—H2B	120.5	N4—C16—C17	117.07 (9)
C3—C2—C1	118.94 (9)	C16—C17—H17	120.6
C3—C2—H2B	120.5	C18—C17—C16	118.75 (9)
C2—C3—H3A	118.7	C18—C17—H17	120.6
C2—C3—C4	122.58 (9)	C17—C18—H18	118.7
C4—C3—H3A	118.7	C17—C18—C19	122.56 (10)
C3—C4—H4B	120.8	C19—C18—H18	118.7
C3—C4—C5	118.37 (9)	C18—C19—H19	121.1
C5—C4—H4B	120.8	C20—C19—C18	117.81 (9)
O2—C5—N1	116.12 (9)	C20—C19—H19	121.1
O2—C5—C4	126.02 (9)	O8—C20—N4	113.69 (9)
N1—C5—C4	117.85 (9)	O8—C20—C19	127.49 (9)
C6—O3—H3	119.1 (16)	N4—C20—C19	118.83 (9)
C10—O4—H4	114.4 (8)	C25—O10—H10	111.5 (8)
C6—N2—H2	118.0 (8)	C25—N5—C21	124.86 (9)
C10—N2—H2	117.5 (8)	C21—N5—H5	117.3 (8)
C10—N2—C6	124.49 (9)	C25—N5—H5	117.8 (8)
O3—C6—N2	114.22 (9)	O9—C21—N5	117.12 (9)
O3—C6—C7	127.87 (9)	O9—C21—C22	126.28 (9)
N2—C6—C7	117.89 (9)	N5—C21—C22	116.60 (8)
C6—C7—H7	120.8	C21—C22—H22	120.4
C8—C7—C6	118.33 (9)	C23—C22—C21	119.17 (9)
C8—C7—H7	120.8	C23—C22—H22	120.4
C7—C8—H8	118.7	C22—C23—H23	118.8
C7—C8—C9	122.55 (10)	C22—C23—C24	122.38 (9)
C9—C8—H8	118.7	C24—C23—H23	118.8
C8—C9—H9	121.0	C23—C24—H24	121.1
C10—C9—C8	117.96 (9)	C25—C24—C23	117.76 (9)
C10—C9—H9	121.0	C25—C24—H24	121.1
O4—C10—N2	113.87 (9)	O10—C25—N5	114.23 (8)
O4—C10—C9	127.39 (9)	O10—C25—C24	126.58 (9)
N2—C10—C9	118.74 (9)	N5—C25—C24	119.19 (9)
C15—O6—H6	112.0 (19)	C30—O12—H12A	112.5 (9)
C11—N3—H3B	116.6 (8)	C26—N6—H6A	116.1 (8)
C15—N3—H3B	118.0 (8)	C30—N6—H6A	119.5 (8)
C15—N3—C11	125.33 (9)	C30—N6—C26	124.46 (9)
O5—C11—N3	116.65 (9)	O11—C26—N6	116.90 (9)
O5—C11—C12	126.15 (9)	O11—C26—C27	125.87 (9)
N3—C11—C12	117.21 (9)	N6—C26—C27	117.24 (9)
C11—C12—H12	120.7	C26—C27—H27	120.5
C13—C12—C11	118.67 (10)	C28—C27—C26	118.93 (10)
C13—C12—H12	120.7	C28—C27—H27	120.5
C12—C13—H13	118.6	C27—C28—H28	118.7
C12—C13—C14	122.76 (10)	C27—C28—C29	122.56 (10)
C14—C13—H13	118.6	C29—C28—H28	118.7
C13—C14—H14	120.8	C28—C29—H29	121.1
C13—C14—C15	118.38 (9)	C30—C29—C28	117.79 (9)
C15—C14—H14	120.8	C30—C29—H29	121.1
O6—C15—N3	115.11 (9)	O12—C30—N6	113.73 (9)
O6—C15—C14	127.23 (9)	O12—C30—C29	127.30 (9)
N3—C15—C14	117.65 (9)	N6—C30—C29	118.97 (9)
			
O1—C1—C2—C3	−179.28 (10)	O7—C16—C17—C18	−178.06 (10)
N1—C1—C2—C3	1.34 (14)	N4—C16—C17—C18	1.06 (14)
C1—N1—C5—O2	−177.46 (9)	C16—N4—C20—O8	178.71 (9)
C1—N1—C5—C4	2.61 (15)	C16—N4—C20—C19	−1.01 (15)
C1—C2—C3—C4	0.39 (16)	C16—C17—C18—C19	−0.44 (17)
C2—C3—C4—C5	−0.75 (16)	C17—C18—C19—C20	−0.91 (17)
C3—C4—C5—O2	179.42 (10)	C18—C19—C20—O8	−178.08 (10)
C3—C4—C5—N1	−0.66 (14)	C18—C19—C20—N4	1.60 (15)
C5—N1—C1—O1	177.61 (9)	C20—N4—C16—O7	178.86 (9)
C5—N1—C1—C2	−2.95 (14)	C20—N4—C16—C17	−0.35 (14)
O3—C6—C7—C8	178.33 (10)	O9—C21—C22—C23	178.40 (10)
N2—C6—C7—C8	0.10 (14)	N5—C21—C22—C23	−1.37 (14)
C6—N2—C10—O4	−178.47 (9)	C21—N5—C25—O10	178.72 (9)
C6—N2—C10—C9	1.86 (15)	C21—N5—C25—C24	−1.47 (15)
C6—C7—C8—C9	1.70 (16)	C21—C22—C23—C24	−0.27 (16)
C7—C8—C9—C10	−1.75 (16)	C22—C23—C24—C25	1.14 (16)
C8—C9—C10—O4	−179.62 (10)	C23—C24—C25—O10	179.46 (9)
C8—C9—C10—N2	0.00 (15)	C23—C24—C25—N5	−0.31 (15)
C10—N2—C6—O3	179.62 (9)	C25—N5—C21—O9	−177.48 (9)
C10—N2—C6—C7	−1.91 (15)	C25—N5—C21—C22	2.31 (14)
O5—C11—C12—C13	−178.89 (11)	O11—C26—C27—C28	−177.73 (11)
N3—C11—C12—C13	0.80 (16)	N6—C26—C27—C28	2.43 (16)
C11—N3—C15—O6	179.01 (9)	C26—N6—C30—O12	179.47 (9)
C11—N3—C15—C14	0.10 (15)	C26—N6—C30—C29	−0.36 (15)
C11—C12—C13—C14	−0.03 (19)	C26—C27—C28—C29	−1.01 (19)
C12—C13—C14—C15	−0.74 (18)	C27—C28—C29—C30	−1.18 (19)
C13—C14—C15—O6	−178.07 (11)	C28—C29—C30—O12	−177.96 (11)
C13—C14—C15—N3	0.70 (15)	C28—C29—C30—N6	1.84 (16)
C15—N3—C11—O5	178.86 (9)	C30—N6—C26—O11	178.32 (9)
C15—N3—C11—C12	−0.86 (15)	C30—N6—C26—C27	−1.83 (15)

### Hydrogen-bond geometry (Å, º)

**Table:**

*D*—H···*A*	*D*—H	H···*A*	*D*···*A*	*D*—H···*A*
O2—H2*A*···O3	0.84 (2)	1.60 (2)	2.4317 (10)	174 (4)
N1—H1···O9^i^	0.88 (1)	1.98 (1)	2.8606 (11)	175 (1)
O3—H3···O2	0.87 (2)	1.57 (2)	2.4317 (10)	176 (3)
O4—H4···O5^ii^	0.981 (14)	1.500 (14)	2.4768 (10)	173.5 (14)
N2—H2···O1^iii^	0.89 (1)	1.89 (1)	2.7554 (11)	166 (1)
O6—H6···O7	0.86 (1)	1.58 (1)	2.4381 (10)	177 (3)
N3—H3*B*···O11^iv^	0.87 (1)	1.95 (1)	2.8155 (11)	170 (1)
O7—H7*A*···O6	0.88 (2)	1.56 (2)	2.4381 (10)	179 (3)
O8—H8*A*···O9^v^	0.91 (1)	1.58 (1)	2.4803 (10)	175 (1)
N4—H4*A*···O10^i^	0.87 (1)	2.07 (1)	2.8999 (11)	161 (1)
O10—H10···O11^iv^	0.95 (1)	1.52 (1)	2.4690 (9)	176 (1)
N5—H5···O2^i^	0.88 (1)	1.90 (1)	2.7699 (11)	167 (1)
O12—H12*A*···O1^iii^	0.90 (1)	1.61 (1)	2.5057 (10)	178 (2)
N6—H6*A*···O5^vi^	0.88 (1)	1.91 (1)	2.7893 (11)	172 (1)

Symmetry codes: (i) −*x*+1, −*y*+1, −*z*+1; (ii) *x*−3/2, −*y*+1/2, *z*−1/2; (iii) *x*−1, *y*, *z*; (iv) −*x*+3/2, *y*+1/2, −*z*+3/2; (v) −*x*, −*y*+1, −*z*+1; (vi) −*x*+3/2, *y*−1/2, −*z*+3/2.
