# Supplementary material for: Synthesis and crystallographic characterization of 6-hydroxy-1,2-dihydropyridin-2-one
Source: Acta Crystallogr E Crystallogr Commun. 2023 Nov 14;79(Pt 12):1147–50. doi: 10.1107/S205698902300974X (PMC10833402; doi:10.1107/S205698902300974X)
Supplement: Supplementary file 3 [file e-79-01147-sup3.docx]

Supplemental Information

for

**Synthesis and crystallographic characterization of 2,6-dihydroxypyridine**

Sara K. Phillips^1^, Savannah G. Brancato^1^, Samantha N. MacMillan^2^, Mark J. Snider^3^, Andrew J. Roering^1*^, and Katherine A. Hicks^1*^

^1^Department of Chemistry, The State University of New York at Cortland, Cortland, New York 13045, United States.

^2^Department of Chemistry and Chemical Biology, Cornell University, Ithaca, New York 14853, United States.

^3^Department of Chemistry, The College of Wooster, Wooster, Ohio 44691, United States.

^*^Corresponding

Tables of Contents

Figure S1: ^1^H NMR spectra of 2,6-dihydroxypyrdine

Figure S2: ^13^C NMR spectra of 2,6-dihydroxypyridine

Figure S3: IR spectra of 2,6-dihydroxypyridine

Figure S1: ^1^H NMR spectra of 2,6-dihydroxypyridine.

Figure S2: ^13^C NMR spectra of 2,6-dihydroxypyridine.


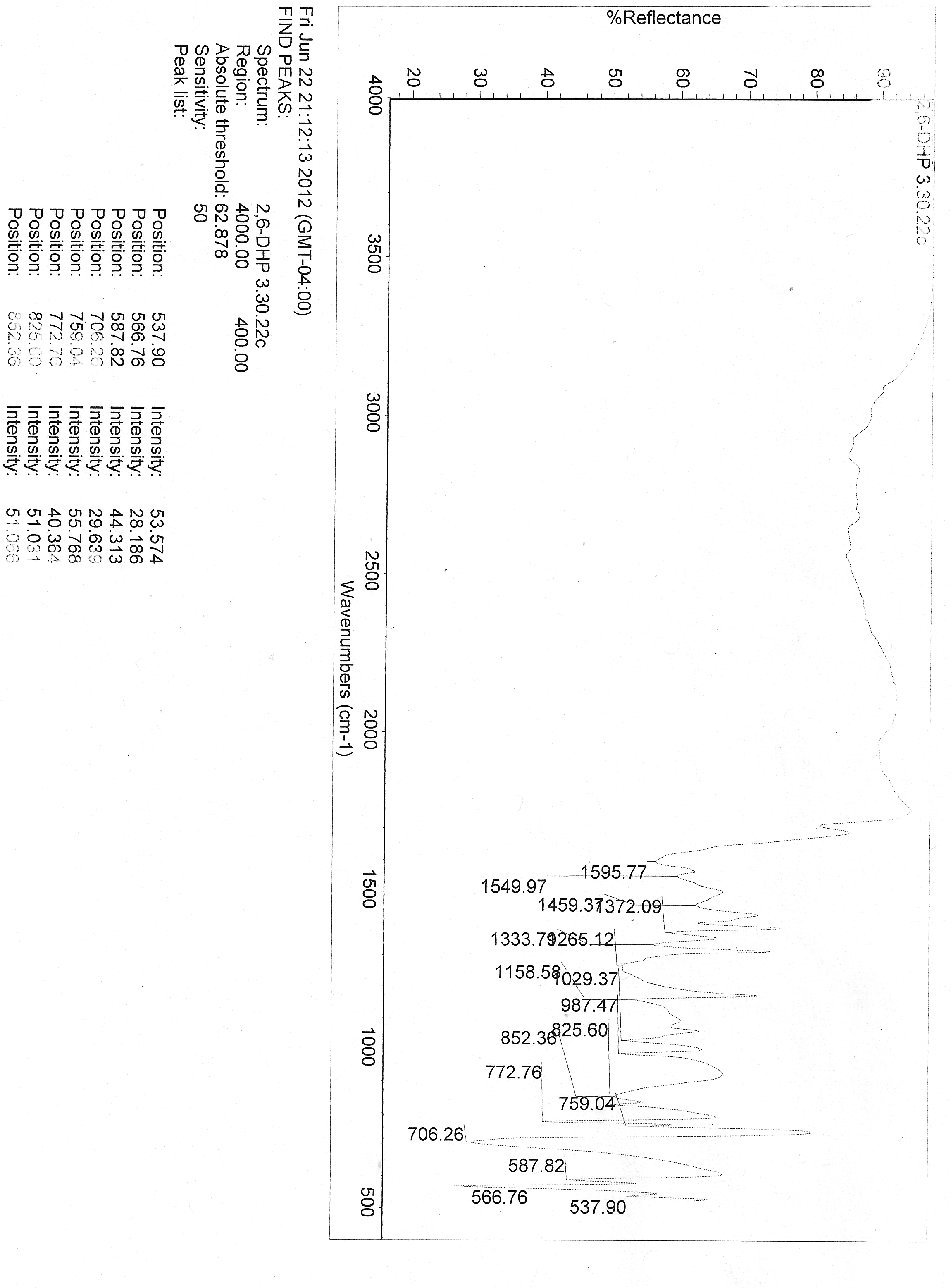


Figure S3. IR spectra of 2,6-dihydroxypyridine.
